# Screen-and-treat program by point-of-care of *Atopobium vaginae* and *Gardnerella vaginalis* in preventing preterm birth (AuTop trial): study protocol for a randomized controlled trial

**DOI:** 10.1186/s13063-015-1000-y

**Published:** 2015-10-19

**Authors:** Florence Bretelle, Florence Fenollar, Karine Baumstarck, Cécile Fortanier, Jean François Cocallemen, Valérie Serazin, Didier Raoult, Pascal Auquier, Sandrine Loubière

**Affiliations:** Department of Gynaecology and Obstetrics, Gynépole, Marseille, Pr Boubli, Hôpital Nord, Assistance Publique-Hôpitaux de Marseille (AP-HM), Aix- Marseille Université, Marseille, France; Aix-Marseille Université, Unité de Recherche sur les Maladies Infectieuses Tropicales et Emergentes, UM63, CNRS 7278, IRD 198, INSERM 1095, Marseille, France; EA3279 Self-perceived Health Assessment Research Unit and Department of Public Health, AP-HM, Aix-Marseille University, Marseille, France; Department of Research and Innovation, Support Unit for clinical research and economic evaluation, Assistance Publique - Hôpitaux de Marseille, Marseille, 13385 France; Hôpital Sainte Marguerite, Assistance Publique - Hôpitaux de Marseille, Marseille cedex 9, France; Service de biologie médicale, CHI Poissy-Saint Germain, Poissy, Cedex France; EA 2493, UFR des sciences de la santé, 78180 Montigny-Le-Bretonneux, France

**Keywords:** Pregnancy, bacterial vaginosis, preterm birth, screen-and-treat strategy, cost-effectiveness analysis, point-of-care, molecular diagnosis, antibiotic treatment, randomized controlled trial, *Atopobium vaginae*, *Gardnerella vaginalis*

## Abstract

**Background:**

International recommendations in favor of screening for vaginal infection in pregnancy are based on heterogeneous criteria. In most developed countries, the diagnosis of bacterial vaginosis is only recommended for women with high-risk of preterm birth. The Nugent score is currently used, but molecular quantification tools have recently been reported with a high sensitivity and specificity. Their value for reducing preterm birth rates and related complications remains unexplored. This trial was designed to assess the cost-effectiveness of a systematic screen-and-treat program based on a point-of-care technique for rapid molecular diagnosis, immediately followed by an appropriate antibiotic treatment, to detect the presence of abnormal vaginal flora (specifically, *Atopobium vaginae* and *Gardnerella vaginalis*) before 20 weeks of gestation in pregnant women in France. We hypothesized that this program would translate into significant reductions in both the rate of preterm births and the medical costs associated with preterm birth.

**Methods/Design:**

A multicenter, open-label randomized controlled trial (RCT) will be conducted in which 20 French obstetrics and gynecology centers will recruit eligible pregnant women at less than 20 weeks gestation with singleton pregnancy and with a low-risk factor for preterm birth. Interventions will include a) an experimental group that will receive a systematic rapid screen-and-treat program from a point-of-care analysis using a molecular quantification method and b) a control group that will receive usual care management. Randomization will be in a 1:1 allocation ratio. The primary endpoint that will be assessed over a period of 12 months will be the incremental cost-effectiveness ratio (ICER) expressed as cost per avoided preterm birth before 37 weeks. Secondary endpoints will include ICER per avoided preterm birth before 24, 28 and 32 weeks, obstetrical outcomes, neonatal outcomes, rates of treatment failure and recurrence episodes for positive women. Uncertainty surrounding these estimates will be addressed using nonparametric bootstrapping and represented using cost-effectiveness acceptability curves. A total of 6,800 pregnant women will be included.

**Discussion:**

This appropriate randomized controlled design will provide insight into the cost-effectiveness and therefore the potential cost savings of a rapid screen-and-treat strategy for molecular abnormal vaginal flora in pregnant women. National and international recommendations could be updated based on the findings of this study.

**Trial registration:**

ClinicalTrials.gov: NCT02288832 (registration date: 30 October 2014); Eudract: 2014-001559-22.

## Background

Prematurity is an important cause of death and disabilities of infants and children [1]. The preterm birth rate was approximately 7.4 % in France in 2010 [2], as observed in other developed countries [3]. The presence of infection and/or inflammation during pregnancy is notably known as a major risk factor for spontaneous preterm birth [4–6]. Pregnant women with bacterial vaginosis (BV) have an increased risk of preterm birth compared to women without BV, and the risk of preterm birth is higher if BV occurs in the early stage of pregnancy [5]. Several bacteria are associated with the diagnosis of BV [7]. However, BV is always associated with the presence of high loads of *Atopobium vaginae* and/or *Gardnerella vaginalis* [8, 9]. Our team previously showed that high vaginal concentrations of *A. vaginae* and/or *G. vaginalis* were associated with a significantly decreased interval between preterm labor and delivery in high-risk pregnancies [10, 11]. Therefore, the control of vaginal flora anomalies (especially *A. vaginae* and/or *G. vaginalis*) in low-risk populations should be considered as an important aim, to see whether their treatments might prevent preterm birth.

In France, the screening (and treatment) of BV is only recommended for women with high-risk of preterm birth [12, 13]. Because BV is asymptomatic in approximately 50 % of cases [14] and early vaginal infection during pregnancy induces higher risk of obstetrical complications, one of the measures to prevent preterm birth should concern a systematic screen-and-treat strategy at the first stage of pregnancy. The standard diagnostic test is the Nugent score [13], which is laborious and not easily reproducible [9]. Such abnormalities have led some authors to propose new diagnostic tools based on molecular biological techniques [7, 9, 15, 16]. The molecular approach based on the quantitative real-time polymerase chain reaction (qPCR) assay appears to be more reproducible and reclassifies a large number of flora rated as intermediate or normal on the Nugent score as true BV [6, 17].

Answering cost-effectiveness questions is a critical step in the translation of technological innovation findings into decision making at the public policy level. Although there is a considerable amount of literature on the economic consequences of preterm birth, little is known about the cost-effectiveness of screen-and-treat strategies for vaginal infection. To date, only one cost-effectiveness analysis of a simple screen-and-treat program for common asymptomatic vaginal infections in pregnancy has been published [18]: it showed cost savings through the first 6 years of life. In this study, the women were tested with a vaginal Gram stain and scoring criteria by the Nugent technique, which does not identify the morphotypes associated with BV such as *A. vaginae,* and the screening was performed in the second trimester of pregnancy, which is probably too late to prevent pregnancy complications.

All these considerations may lead to questions on the cost effectiveness of a systematic screen-and-treat program during the first trimester for flora vaginal anomalies among pregnant women with a low-risk of preterm birth, taking into account advances in diagnostic tools, bacteria species targeted and antibacterial treatments. For this purpose, a multicenter, randomized controlled study was designated to assess the cost effectiveness of the innovative screening for *A. vaginae* and *G. vaginalis* vaginal portage using a molecular quantification method by point-of-care with an appropriate treatment for positive cases, compared to a usual care strategy in pregnant women at less than 20 weeks of gestation. Several obstetrical and neonatal secondary outcomes are also considered, as well as rates of treatment failure and recurrence episodes.

## Methods/Design

### Design

**Fig. 1:**
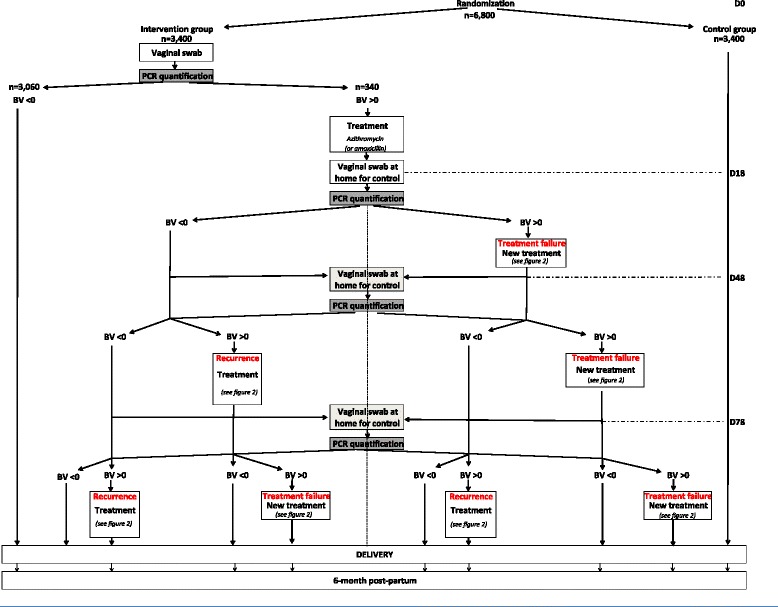
Schema of timing and phasing - AuTop Study

A multicenter, open-label randomized controlled, two-parallel group study was designed in which pregnant women who attend prenatal care consultations before 20 weeks’ gestation at French obstetrics and gynecology centers are randomized between two management strategies: a systematic vaginosis screen-and-treat strategy (experimental group) and usual care management (control group). The study protocol was designed using the recommendations of the Consolidated Standards of Reporting Trials (CONSORT) statement and according to the guidelines of cost-effectiveness studies of the French Health Authority [Haute Autorité en Santé, Choix méthodologiques pour l’évaluation économique à la HAS http://www.has-sante.fr/portail/jcms/c_1120711/choix-methodologiques-pour-l-evaluation-economique-a-la-has]. Timing and phasing after eligibility checks are shown in Fig. 1.

### Partners

This work is supported by an institutional grant from the French 2014 National Program of Cost-effectiveness Research (Programme de Recherche Médico-Economique, FINESS number 130789049). The recruiting will take place in 20 French obstetrics and gynecology centers. The molecular analyses will be performed in two point-of-care laboratories. The methodological support will be provided by the Clinical and Cost-Effectiveness Research Unit (Assistance Publique - Hôpitaux de Marseille, AP-HM, France), and the Clinical Investigation Unit (Centre d’Investigation Clinique, AP-HM, France). The central pharmacy of AP-HM is in charge of the assignment, allocation and delivery of the devices. All the details are provided in Table 1.

**Table 1:** French partners

Gynecologists	Center/department
Pr Florence Bretelle	Coordinating investigator. Public academic teaching hospital Nord, Marseille
Dr Hélène Heckenroth	Public academic teaching hospital La Conception, Marseille
Dr Raoul Desbriere	Private Hospital Saint Joseph, Marseille
Dr Nadia Slim	Private Hospital Bouchard, Marseille
Dr Nawal Chenni-Asselah	Public hospital, Aubagne
Dr Xavier Danoy	Public academic hospital, Aix-en-Provence
Dr Franck Mauviel	Public academic hospital, Toulon Sainte Musse
Pr André Bongain	Public academic teaching hospital, Nice
Pr Pierre Mares	Public academic teaching hospital, Caremeau
Pr Patrick Rozenberg	Public academic hospital, Poissy-Saint-Germain
Dr Thomas Schmitz	Public academic teaching hospital Robert Debré, Paris
Pr Alexandra Benachi	Public academic teaching hospital Antoine Béclère, Clamart
Pr Marie-Victoire Senat	Public academic teaching hospital Kremlin-Bicêtre, Kremlin-Bicêtre
Pr Bassam Haddad	Public academic hospital, Créteil
Dr Jean-Pierre Ménard	Protection Maternelle Infantile, Val de Marne
Pr Gilles Kayem	Public academic teaching hospital Armand Trousseau, Paris
Pr Loic Sentilhes	Public academic teaching hospital, Angers
Pr Céline Chauleur	Public academic teaching hospital, Saint-Etienne
Dr Jean-Luc Volumenie	Public academic teaching hospital, Martinique
Dr Philippe Kadhel	Public academic teaching hospital, Pointe-à-Pitre
Clinical microbiologists	Center/department
Pr Florence Fenollar	Fédération de Microbiologie Clinique. Public academic teaching hospital la Timone, Marseille
Dr Valérie Serazin	Service de Biologie Médicale - UF de Biologie moléculaire. Public general hospital, CHI Poissy St Germain
Methodology team	Center/department
Pr Pascal Auquier	Public health, public academic teaching hospital, Marseille
Sandrine Loubière & Cécile Fortanier	Health economy, public academic teaching hospital, Marseille
Dr Karine Baumstarck	Clinical research unit, public academic teaching hospital, Marseille
Dr Nathalie Lesavre	Clinical investigation center, public academic teaching hospital, Marseille
Dr Stéphane Honoré & Dr Anita Cohen	Pôle Pharmacie, public academic teaching hospital, Marseille

#### Inclusion and exclusion criteria

The details of the inclusion and exclusion criteria are provided in Table 2. The main inclusion criteria are that women must have less than 20 weeks of gestation, with singleton pregnancy, they must be symptomatic or nonsymptomatic with regard to their diagnosis of BV, and they must not have high-risk factors of preterm birth. The main exclusion criteria are having known conditions at the time of recruitment that have either increased risk of spontaneous preterm birth (previous preterm birth, uterine malformation, or multiple pregnancy) or that may need preterm delivery due to medical indication: hypertension, diabetes, fetal malformation, increased risk for preeclampsia (or other conditions that the investigators may consider).

**Table 2:** Selection criteria

Inclusion criteria
Woman ≥ 18 years of age
Woman less than 20 weeks of gestation
Woman with singleton pregnancy
Woman without history of preterm birth or late miscarriage
Woman with low-risk factor of preterm birth (absence of diabetes, systemic lupus erythematosus, treated hypertension, fetal malformation, cervical conization, or multiple pregnancy)
Woman affiliated to or beneficiary of a social security system
Woman who have signed written informed consent
Exclusion criteria
Woman more than 20 weeks of gestation
Minor woman or woman deprived of their freedom by a court/administrative decision or woman under legal protection
Woman who present high-risk factor of preterm birth or late miscarriage
Woman with extrauterine pregnancy
Woman with non-evolutive pregnancy
Woman who have received antibiotic treatment in the week before inclusion
Woman misunderstanding the written and spoken French language
Subject participating in another biomedical research protocol

### Interventions

#### Experimental group: screen-and-treat strategy

**Fig. 2:**
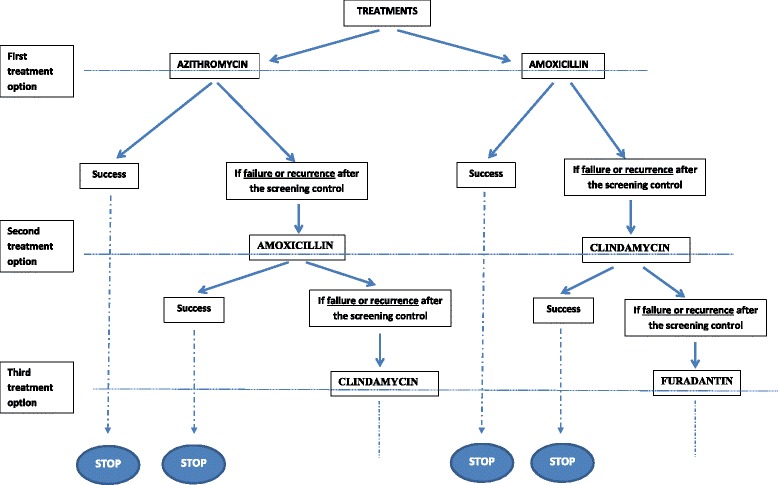
Schema of treatment algorithm alongside the trial - AuTop Study

Pregnant women assigned to the intervention group are asked for a self-collected vaginal swab at randomization (Time 0). The self-collected vaginal swab has previously been demonstrated to have high validity and reliability compared to a practitioner-collected swab [17]. The swab will be immediately tested for *A. vaginae* and *G. vaginalis* using a systematic point-of-care screening test. Quantitative molecular analyses will be performed in laboratories that have experienced and national accreditation to realize point-of-care techniques. The quantitative real-time PCR (qPCR) method used to diagnose BV was previously described [11]. The result is reported as copies of microorganism DNA per 1 mL of vaginal suspension [9]. According to previous works [9, 11], BV will be defined by an *A. vaginae* load ≥ 10^8^ copies/mL and/or a *G. vaginalis* load ≥ 10^9^ copies/mL. The conclusion will be feedback of the positive or negative test result to the practitioner within less than 24 hours. In cases of positive diagnosis, the pregnant woman will be referred to an obstetrician, and an appropriate antibiotic treatment will be provided. The first intention for treatment of *A. vaginae* and *G. vaginalis* will be azithromycin (single dose, 1 g at day 1 and 1 g at day 3). The second choice for antibiotic treatment will be amoxicillin 2 g per day during 7 days in cases of known intolerance of azithromicin. The following procedure for women who are diagnosed positive is to perform a series of three successive screening controls until 28 weeks to detect either the failure of antibiotic treatment or a recurrence (that is, reappearance of bacteria from a control vaginal swab after therapy is stopped) (see Fig. 1). In cases of recurrence or treatment failure, a new antibiotic treatment will be provided, based on protocol guidelines (see Fig. 2). For women with a first negative diagnosis, the usual care management will be proposed.

#### Control group: usual care management

No systematic vaginosis screening will be performed on the women assigned to the control group according to national and international guidelines [13, 19]. Usual pregnancy care includes 6 to 8 obstetrical consultations, no systematic vaginal swab, three ultrasound scans, 1st trimester Down syndrome screening and blood sampling for toxoplasmosis, syphilis, rubella, and complete blood group testing.

### Recruitment

Eligible women will be invited to take part in the study during a routine prenatal consultation planned in the first trimester of their pregnancy. The women who meet all the inclusion criteria will be randomized into one of the two groups after completing the informed consent form.

### Randomization

Computer-generated randomized lists were drawn up before the beginning of the study, using a permuted block design, under the responsibility of the clinical research unit (AP-HM). The randomization was stratified by center (1:1 allocation ratio).

### Endpoints

#### Primary endpoint

The primary endpoint is the incremental cost-effectiveness ratio (ICER), expressed as the extra cost per additional preterm birth avoided before 37 weeks. The effectiveness criterion has been discussed and consensually approved by all the study’s main partners (gynecologist coordinator and co-coordinators, biologists, health economist and methodologist). We can assume that this intermediary criterion properly reflects short- and long-term prognosis of the children [20, 21].

#### Secondary endpoints

The secondary endpoints are as follows:

1. ICER per preterm birth avoided before 26, 28, 32 and 34 weeks.
2. Obstetrical outcomes: rates of preterm birth before 26, 28, 32, 34, and 37 weeks of gestation, spontaneous abortion (between 14-22 weeks), late miscarriage (between 22-24 weeks), premature rupture of membranes, severe intrauterine growth restriction, preterm labor, duration of the woman’s hospitalization (that is hospitalization for delivery or preterm labor and potential previous hospitalizations due to gynecologic complications during pregnancy).
3. Neonatal outcomes: neonatal mortality, neonatal morbidity (respiratory distress syndrome, bronchopulmonary dysplasia, necrotizing enterocolitis, periventricular leukomalacia), transfer to a neonatal intensive care unit (duration), mechanical ventilation (duration), congenital anomalies and duration of the newborn’s hospitalization.
4. Treatment effectiveness: rate of recurrence (defined as a positive control vaginal swab using qPCR after the negativation of a precedent control vaginal swab), rate of treatment failure (defined as *A. vaginae* > 10^8^ copies/mL and/or a *G. vaginalis* load ≥ 10^9^ copies/mL from a control vaginal swab) and side effects associated with treatment.
5. Other health care utilization: all the mother’s use of health care during the whole study period (for example, gynecologists and general practitioner consultations, hospital admission, clinical examinations and medications), as well as health care for the newborn (including neonatal care, re-hospitalization, medications, planned and non-planned consultations with pediatric practitioner or other specialists).

### Data collection and follow-up

All data will be recorded from an electronic case report form (eCRF) specifically elaborated for the study (eCRF CleanWEB, Telemedicine Technologies S.A.S., www.tentelemed.com, 2015) and will be recorded at four specific study’s times as follows: randomization (T0), baseline assessment (T1), delivery (T2), and at 6 months after delivery (T3). All assessments are based either on medical files (pregnancy and delivery characteristics, obstetrical and neonatal outcomes), face-to-face questionnaires (smoking and alcohol habits, personal hygiene, pregnancies history or symptoms, and concomitant treatments such as treatment with pessary or progesterone), phone calls (to collect data on vaginal symptoms or potential side-effects of antibiotic) or self-report (health outcomes of their infant and health service use during the 6 months following the initial hospitalization). Details of the data recorded at the different times of the study are given in Table 3.

**Table 3:** Data collection, instruments and assessment times

At randomization (T0)	Data on the health status of the participant and pregnancy characteristics will be collected from medical files of the practitioners. A face-to-face questionnaire will be also completed by all women and filled out by the midwife/obstetrician to collect specific data on demographics characteristics, smoking and alcohol habits, previous pregnancies and personal hygiene. The first vaginal swab will be collected by the practitioner and send to one of the two point-of-care laboratories associated to the study.
At baseline assessment – (during screening and treatment phases - T1)	Subsequent vaginal swabs will be realized during either routine pregnancy consultation or at the woman’s home depending on each participant schedule. In this later case, the sample will be sent by the women to the referent POC laboratory using a stamped, self-addressed envelope. Symptoms and potential side effects of antibiotics will be collected via a telephone interview. Participants will be informed via a phone call for subsequent vaginal swabs and treatment intake if needed.
At delivery (T2)	All relevant clinical and obstetrical outcomes during pregnancy will be collected from the medical files. To complete data collection, a face-to-face interview with women around delivery phase will be scheduled. All relevant data such as pregnancy complications, hospitalizations, delivery characteristics (including birth weight, terms at delivery, or fetal death) will be collected.
At 6 months after delivery (T3)	Participants will be provided with a questionnaire, on which they will be asked to record all health outcomes of their infant and associated health service use.

### Pharmaceutical aspects

Experimental drugs, azithromycin 250 mg oral tablets (ZITHROMAX™) and amoxicillin 500 mg oral capsules (CLAMOXYL™), will be packaged and labeled by the “Unité d’Expertise Pharmaceutique et Recherche Biomedicale” pharmaceutical unit of the Hospital Pharmacy of AP-HM and distributed to the dispensing hospital pharmacy of each investigating center. At the end of the study, used and unused treatments will be returned to the dispensing pharmacy and destroyed.

### Sample size

The sample size was calculated from the expected differential ICER per preterm birth avoided between the two groups. In accordance with Briggs [22], the following hypothesis is stated: with an expected incremental rate of preterm birth of 1.3 % (4.3 % in the control group [23] and 3.0 % in the experimental group), an expected incremental cost of 230 euros (including cost of initial and following point-of-care tests and cost of treatments for 10 % of the women [24]), and an expected differential ICER at 22,500 euros corresponding to the avoided cost of a preterm birth before 37 weeks [25], with an 80 % statistical power and a threshold for statistical significance set at a *P* value of 0.05, and assuming that a potential 20 % of patients will be lost to follow-up, these calculations showed that 6,800 patients are needed (3,400 per group). Considering the potential of inclusion of each participating center, the inclusion duration will be planned over a 12-month period. The maximal period of participation for the included women is 12 months.

### Statistical analysis

The data will be analyzed using SPSS version 20.0 software. Statistical significance is defined as *P* < 0.05. The methodology will be based on the Consolidated Standards of Reporting Trials Statement (CONSORT, http://www.consort-statement.org/consort-statement/) [26]. The full analysis population (including all subjects who will be randomized and will be at least evaluated at baseline) will be used in the primary analysis and the per protocol population (including all subjects who will be randomized and will not have major protocol deviations) will be used in the secondary analysis to assess the reliability of the results. Finally, missing data will be handled where possible using multiple imputations, and a sensitivity analysis will be performed. No interim analysis is planned.

The normality of the parameters will be estimated using frequency histograms and the Shapiro test. In case of nonparametric distribution, the data will be log transformed to obtain a normal distribution or nonparametric bootstrapping performed for cost data. In accordance with the distribution of the parameters, the baseline parameters will be presented separately for the control group and the experimental group: mean (standard deviation) or median (interquartile range) for continuous variables, proportions for categorical variables. Then, data will be compared between the two groups using Student’s t-test for continuous variables (durations), and chi-square or Fisher’s exact tests for categorical variables.

### Cost-effectiveness analysis

Incremental cost-effectiveness ratios (ICER) will be used to compare the cost and effectiveness of the experimental strategy with usual care. The ICER is the ratio of the difference between groups in costs to the difference in effectiveness. The difference in effectiveness equals the number of preterm births averted and is calculated as the number of preterm births in the screening group minus the number of preterm births in the control group. Thus, the ICER provides information on the potential acceptability of the intervention for decision makers. The costs perspective taken in our economic analysis is that of the healthcare payer. The time horizon started from the first prenatal consultation before the 20 weeks of gestation and ended at 6-months of age or death. The healthcare costs included are those that are likely to differ across the intervention and control groups. In our study, these costs are those associated with: screening using the point-of-care procedure (quantitative molecular analysis), control vaginal swabs for positive women, antibiotic treatments, antenatal hospital admissions, physicians’ consultations, management of complications, as well as neonatal costs for full term infants and preterm infants. Unit costs for health service use will be estimated using data from the French National Hospital Database (Programme de médicalisation des systèmes d’information, PMSI) and the National Tariff. Treatment costs were obtained from the French register of pharmaceutical specialties, an online database of information on healthcare products. The cost of the POC analysis using quantitative real-time PCR (qPCR) will be calculated using micro-costing technique, which is particularly well suited to evaluating the cost of an emerging technique [27]. The volume of resources used will be determined by direct observation of each stage of each testing procedure. Fixed and variable costs will be included. All resources will be valued in 2015/2016 euros, and the 12-month trial means there is no requirement to apply discounting. As the cost of preterm birth differs significantly according to the gestational age at birth [28–30], we will attempt to address this issue by calculating an ICER for specific birth gestational ages. Probabilistic sensitivity analyses, using the non-parametric bootstrap method, will be carried out to generate mean expected ICERs and to determine whether uncertainty or variation in the data used affect the ICERs [31]. In addition, cost-effectiveness acceptability curves were constructed to represent decision uncertainty surrounding cost-effectiveness estimates [32].

### Ethical aspects, laws and regulations

The study will be conducted in accordance with the Helsinki declaration and the French laws and regulations (Code de la Santé Publique, article L.1121-1/Loi de Santé Publique n°2004-806 du 9 Août 2004 relative à la politique de santé publique et ses décrets d’application du 27 Août 2006) and the International Conference on Harmonization (ICH) E6 Guideline for Good Clinical Practice. Regulatory monitoring will be performed by the sponsor. French ethics committee and French drug and device regulation agency approved conduct of the AuTop study at all sites (registered number respectively: Comité de Protection des Personnes Sud Méditerranée, reference number 14.026 and Agence Nationale de Sécurité du Médicament, reference number 140500A-41). This trial was registered into the government clinical trials under the identifier number NCT02288832 (ClinicalTrials.gov). Written Informed consent will be obtained from all subjects before inclusion.

## Discussion

This is the first large randomized controlled trial assessing the cost-effectiveness of a screen-and-treat program of molecular flora vaginal anomalies during the first trimester in pregnant women with low risk of preterm birth. This study was designed specifically to inform healthcare decision making, in an international context where the diagnosis of BV in pregnant women and its subsequent management care are controversial [33–37]. Several reasons could be stressed to explain these diverging opinions.

Firstly, there was a real lack of accuracy of diagnostic tools at the time of some studies. Most of the studies cited earlier focused on the Nugent score, which has some limitations. It must be performed on a fresh swab, and any delay in transporting the swab makes the test difficult to perform, its scoring requires experienced microbiologists to ensure consistency, and some pathogens associated with BV are not identified by such a technique, in particular *A. vaginae* [9]. Recent studies have demonstrated that molecular biology techniques have both higher sensitivity and specificity for the diagnosis of BV [7, 9, 15, 16] compared to the Nugent score and can detect several bacterial series for BV.

The second point concerns the appropriateness of antibiotic treatment [13, 19]. The nature of the antibiotics (such as metronidazole or clindamycin, pro- or prebiotics) and their modes of administration (oral versus vaginal) vary considerably between existing studies. Few studies have controlled for treatment efficacy [38]. In addition, although the frequency of recurrences after antibiotic treatment is high (from 28 % to 50 %), depending on the nature of the treatment and the length of time from its intake [39], most of studies did not consider this risk of recurrence. In the present project, we propose to use azithromycin because of its effectiveness on *A. vaginae* and *G vaginalis* with a lifetime that allows us to reduce the treatment duration and to increase the adherence rate [40].

The third point concerns the delay between BV and treatment. In cases of late diagnosis, and therefore delayed treatment, BV has already been established and can lead to obstetrical complications involving preterm birth [41]. In this present study, we have considered that the point-of-care test will be an interesting approach to minimize the feedback of the vaginal swab to the practitioner in order to rapidly prescribe the antibiotic treatment in cases where tests are positive. In our trial, the short window for providing a diagnostic result also appears essential for minimizing the number of women lost to follow-up who may benefit from an early antibiotic treatment. Indeed, this rate of “lost to follow-up patients” is important in this disease where asymptomatic disease, anxiety and low socioeconomic status are clearly identified risk factors [9].

Finally, the gestational age at inclusion is the greatest limitation of previous published studies. Due to the lack of data concerning the early phase of pregnancy, the authors may have underestimated the effectiveness of a universal screen-and-treat program in reducing the rate of “very preterm birth” (28 to 32 weeks). These subdivisions into “very preterm” and “moderate” or “late preterm” (that is, 32 to less than 37 completed weeks of gestation) are important since decreasing gestational age is associated with increasing mortality, intensity of neonatal care required, and hence increasing costs [42].

The main purpose of AuTop trial is to support at a nationwide level the feasibility, acceptability, and cost-savings of a routine point-of-care molecular diagnosis at the first stage of pregnancy. Although it is well known that the consequences in terms of morbidity and mortality of preterm birth are important and entail a significant economic burden, several studies have demonstrated that a large share of the incremental cost per surviving preterm infant is represented by neonatal care [43, 44]. The average length of this neonatal care can vary from 3 days to 6 months for very preterm births (including potential immediate re-hospitalization, programmed consultations and bronchiolitis prevention measures). We then decided to focus on this time horizon for the cost-effectiveness analysis.

In Kiss et al. [23], the prevalence of BV was around 7 % in their study population (which included women without subjective complaints, that is, contractions, vaginal bleeding, or symptoms suggestive of vaginal infection, or women with multiple pregnancies), with less than 2.3 % having an obstetric history at inclusion. It is well known that the ethnic origin influences the prevalence of bacterial vaginosis in pregnancy. In Kiss’s RCT, the population was 98 % white ethnic origin. In our study population, we will expected less than 65 % for the white ethnic group, based on our previous RTC conducted in the same French centers [11]. Consequently, we think that a prevalence of BV of 10 % in a population with or without clinical symptoms of BV and well balanced in terms of ethnic origins is not so optimistic. Our hypothesis of a decrease in the rate of preterm births from 4.3 % to 3.0 % seems to be a small expected gain in the rate of preterm births avoided. In fact, a mean difference of 1.3 % is actually a very large difference for pregnant women and decision makers, and should be sufficient to prevent major healthcare expenditure. Decision analysis is particularly useful when the difference in outcomes between strategies is small, and can provide insight into the costs contributing to the public decision-making process.

Given the need for scientific evidence (in terms of both efficacy and economic) regarding bacterial vaginosis screening in a population with low-risk factors for preterm birth, our analysis should be useful for clinicians and other healthcare decision makers involved in managing care of pregnant women.

## Trial status

At the time of manuscript submission, the status of the trial is yet recruiting. The inclusion of participants started in March 2015.

## Abbreviations

AAP: American Academy of Pediatrics
ACOG: American Congress of Obstetricians and Gynecologists
ANSM: Agence Nationale de Sécurité du Médicament
AP-HM: Assistance Publique-Hôpitaux de Marseille
BV: bacterial vaginosis
CDC: Centre for Disease and Controls
CONSORT: Consolidated Standards of Reporting Trials
DNA: deoxyribonucleic acid
eCRF: electronic case report form
HAS: Haute Autorité en Santé - French National Authority for Health
ICER: incremental cost effectiveness ratio
ICH: International Conference on Harmonization
PCR: polymerase chain reaction
SPSS: Statistical Package for the Social Sciences
PMSI: Programme de médicalisation des systèmes d’information
RCT: randomized controlled trial
