# Erratum to: ‘Screen-and-treat program by point-of-care of Atopobium vaginae and Gardnerella vaginalis in preventing preterm birth (AuTop trial): study protocol for a randomized controlled trial’

**DOI:** 10.1186/s13063-016-1219-2

**Published:** 2016-02-12

**Authors:** Florence Bretelle, Florence Fenollar, Karine Baumstarck, Cécile Fortanier, Jean François Cocallemen, Valérie Serazin, Didier Raoult, Pascal Auquier, Sandrine Loubière, the Groupe de Recherche en Obstétrique et Gynécologie (GROG)

**Affiliations:** Department of Gynaecology and Obstetrics, Gynépole, Marseille, Pr Boubli, Hôpital Nord, Assistance Publique-Hôpitaux de Marseille, AMU, Aix- Marseille Université, Marseille, France; Aix-Marseille Université, Unité de Recherche sur les Maladies Infectieuses Tropicales et Emergentes, UM63, CNRS 7278, IRD 198, INSERM 1095, Marseille, France; EA3279 Self-perceived Health Assessment Research Unit and Department of Public Health, AP-HM, Aix-Marseille University, Marseille, France; Department of Research and Innovation, Support Unit for clinical research and economic evaluation, Assistance Publique – Hôpitaux de Marseille, Marseille, 13385 France; Hôpital Sainte Marguerite, Assistance Publique – Hôpitaux de Marseille, Marseille cedex 9, France; Service de biologie médicale, CHI Poissy-Saint Germain, Poissy Cedex, France - EA 2493, UFR des sciences de la santé, 78180 Montigny-Le-Bretonneux, France

After publication of this work [1], we noted that we failed to include the complete list of all coauthors. The full list of authors has now been updated. The Authors’ contributions, Acknowledgements and Competing interests section have been modified accordingly. We are publishing this erratum to update the author list, which is as follows:

Florence Bretelle, Florence Fenollar, Karine Baumstarck, Cécile Fortanier, Jean François Cocallemen, Valérie Serazin, Didier Raoult, Pascal Auquier, Sandrine Loubière, and the Groupe de Recherche en Obstétrique et Gynécologie.
